# Intrauterine Limb Ischemia in Patient Heterozygous for the 677C>T) RS1801133 (Polymorphism of Methylenetetrahydrofolate Reductase MTHR Gene

**DOI:** 10.1155/2022/2701548

**Published:** 2022-10-21

**Authors:** Ahmad Al Hammouri, Rami A. Misk, Hamza Abumunshar, Fawzy M. Abunejma, Tasnim S. Idrees, Mahmoud Abu Arqoub, Deiaa Malhis, Abdullah Shroof, Tareq Z. Alzughayyar

**Affiliations:** ^1^College of Medicine and Health Sciences, Palestine Polytechnic University, Hebron, State of Palestine; ^2^Department of Radiology, Al-Ahli Hospital, Hebron, State of Palestine; ^3^Department of Plastic and Reconstructive Surgery, Hamad General Hospital, Hamad Medical Corporation, Doha, Qatar; ^4^Department of Pediatrics, Al-Ahli Hospital, Hebron, State of Palestine

## Abstract

**Background:**

Intrauterine arterial thrombosis is extremely rare. Multiple inherited coagulopathies were found to be associated with thrombophilia and an increased risk of intrauterine arterial thrombosis. Methylenetetrahydrofolate reductase MTHFR (C667T) polymorphism was found to be associated with mild hyper-homocysteinemia, which, in turn, can promote thrombotic complications.

**Materials and Methods:**

We reported a case of intrauterine upper limb ischemia in a neonate who was found to be heterozygous for the 677C > T polymorphism of the MTHFR gene despite the dispute regarding its clinical significance as a risk of arterial thrombosis. We also reviewed the literature and summarized the clinical features, treatment, and prognosis of similar cases. *Case Presentation*. We reported a full-term female, born by normal spontaneous vaginal delivery who was found to have a swollen, blue left upper limb in the delivery room. Left upper limb computed tomography angiography (CTA) revealed left subclavian artery thrombosis. Investigations for the risk revealed heterozygosity for the MTHFR (C667T) polymorphism. Left upper limb amputation was done after the failure of medical management.

**Conclusion:**

Despite the conflict about whether heterozygosity for MTHFR (C667T) polymorphism increases the risk of arterial thrombosis or not, there are few cases in the literature presented with intrauterine upper limb ischemia and were found to be heterozygous for the mutation. We recommend investigating neonates and their parents for complete thrombophilia mutations when they present with unusual vascular occlusion sites as newborns.

## 1. Introduction

Intrauterine limb ischemia is considered very rare with a limited number of cases being reported in the literature [1–4]. Overall congenital upper limb circulatory deficiency can affect approximately 7 out of every 10000 births [5]. It must be discovered and dealt with promptly in order to preserve limb function and restore perfusion as it may lead to limb amputation in many cases [4, 6].

Intrauterine limb ischemia must be differentiated from neonatal limb ischemia, which occurs after birth mostly due to catheterization, sepsis, or coagulopathy [1–4]. On the other hand, intrauterine upper limb ischemia may result due to intrauterine compression by amniotic bands, oligohydramnios, mal-presentation of the fetus with limb prolapse and umbilical cord entanglement, or as a result of thromboembolic events due to embolization from infarcted placental parts or thrombosis due to coagulopathies [2–8].

Despite the debate regarding the clinical significance of MTHFR gene polymorphism as a risk factor for arterial thrombosis [9], McKasson and Golomb reported congenital upper limb ischemia in two newborns who were found to be homozygous for the 677C > T polymorphism of the MTHFR gene [10]. Other articles reported limb ischemia in heterozygotes for 677C > T polymorphism [11–13].

We present a case of intrauterine upper limb ischemia in a neonate who was found to be heterozygous for the 677C > T polymorphism of the MTHFR gene.

## 2. Case presentation

A full-term 3665-g female was born to a 23-year-old mother (G2P2A0) by NSVD after an uneventful pregnancy with cephalic presentation. Apgar scores were 8 and 9 at one and five minutes, respectively. Delivery was uncomplicated, no history of shoulder dystocia or any trauma during delivery. Placenta gross examination was unremarkable.

**Figure 1:**
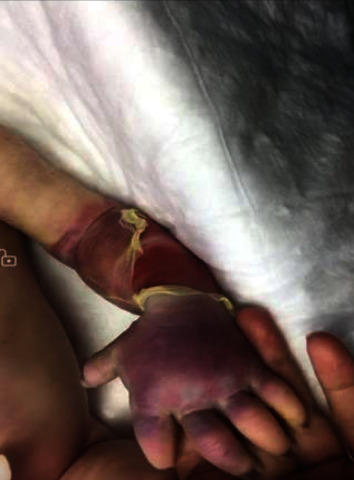
A photograph that shows swelling, cyanosis, and skin maceration in the left forearm and hand of our patient.

Prenatal and antenatal histories were uneventful with no maternal history of preeclampsia, gestational diabetes, infections, or hyper-coagulopathy. Her father was a 25-year-old with unremarkable medical history. No history of parental consanguinity was found. In the delivery room, the neonate was found to have a swollen, blue left upper limb with skin maceration. As a result, the baby was admitted to the Neonatal Intensive Care Unit (NICU). Physical examination there revealed below elbow cyanosis and skin maceration in the left upper limb (Figure 1). The left upper limb was cold compared to the right one. Radial, ulnar, and brachial pulses could not be felt with no spontaneous movements, Moro reflex, or grasp reflex on the left side. Otherwise, the examination was unremarkable including growth parameters.

Initial laboratory evaluation revealed a normal complete blood count and coagulation profile. Coombs test was negative, CRP was 17.7, and blood cultures showed no growth. Chest and upper limb x-rays revealed no fractures.

**Figure 2:**
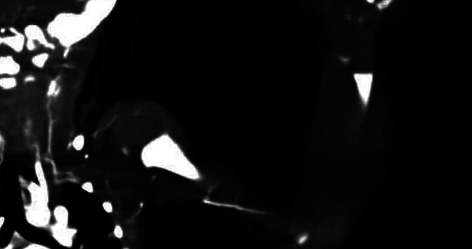
CTA shows a contrast filling defect in the left subclavian artery, starting about 2 cm from the origin of the aortic arch as a result of left subclavian artery thrombosis.

Doppler ultrasound for the left upper limb at 2 hours of age revealed no evidence of blood flow through the subclavian or brachial arteries. Left upper limb computed tomography angiography (CTA) was done at age of 6 hours and was significant for a thrombus occluding most of the subclavian artery, starting about 2 cm from the origin from the aortic arch. There was a contrast filling the proximal 1 cm of the axillary artery mostly from collaterals with a complete cutoff of the contrast thereafter until the level of the midarm, after which arterial contrast filling resumed until the level of the elbow with no contrast flow beyond the level of the elbow joint, consistent with a diagnosis of left subclavian artery thrombosis that is causing upper limb ischemia (Figure 2).

Echocardiography showed no evidence of congenital heart disease. Carotid Doppler was normal with patent blood flow bilaterally. Computed tomography angiography (CTA) for the aorta and its branches revealed left subclavian artery thrombosis as we previously mentioned. Otherwise, the aorta, inferior vena cava, umbilical, and renal vessels were normal. Trans-fontanel ultrasound showed no hemorrhage.

A thrombophilia screen revealed normal levels of protein C, protein S, and antithrombin. Lupus antiphospholipids and anticardiolipin antibodies were negative. Screening for genetic procoagulant mutations was done, including DNA-based assays for ACE I/D, PAI-1 4 G/5G, Factor XIII Val34Leu, HPA1 (1a/1b), Factor V Leiden, and prothrombin G20210A were all normal.

She was found to be heterozygous for methylenetetrahydrofolate reductase MTHFR (C667T) polymorphism with normal levels of homocysteine in her blood [7, 11]. Heparin drip was started at 4 hours of age (28 units/kg/hour), and adjusted to keep activated partial thromboplastin time (aPTT) at a target of 2-3*x* normal. Shifted to low-molecular-weight heparin (Enoxaparin) (2 mg/kg subcutaneously BID) at 46 hours of age for 3 days.

**Figure 3:**
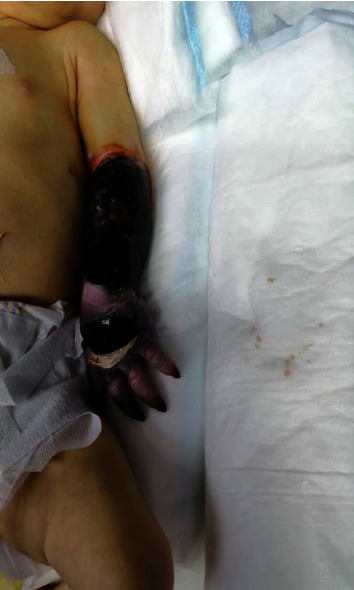
A photograph that shows left forearm and hand gangrene in our patient after the failure of medical management.

Her limb showed no improvement and developed wet gangrene (Figure 3). Orthopedic and vascular surgeons were consulted. Left upper limb amputation was done after the failure of medical management. The neonate then left the hospital on low-molecular-weight-heparin (LMWH).

## 3. Discussion

Symptomatic arterial thrombosis in neonates is extremely rare. It can affect nearly 1 in every 20000–30000 births and contributes to approximately 2.4–6.8 per 1000 neonatal intensive care unit (NICU) admissions according to the literature [1, 14, 15]. It occurs due to iatrogenic causes (catheterization) in 90% of cases, but can also be attributed to sepsis and coagulopathies [1–4].

Intrauterine arterial thrombosis is even rarer than neonatal arterial thrombosis. It occurs mostly due to intrauterine compression by amniotic bands, oligohydramnios, or umbilical cord compression. It may also occur due to embolization from infarcted placental parts, hyper-viscosity due to polycythemia, or thrombosis due to inherited thrombophilia [2–8].

Usually, affected newborn presents with pallor, cyanosis, swelling, coldness, macerations, absent peripheral pulses, prolonged capillary refill time (CTR), and possibly necrosis of the involved limb during the first hours of life. [10–13].

Multiple inherited coagulopathies were found to be associated with thrombophilia and an increased risk of intrauterine arterial thrombosis. Protein C deficiency, protein S deficiency, Factor V mutation, prothrombotic polymorphisms, Factor II G2021A, and the homozygous TT genotype of the methylenetetrahydrofolate reductase (MTHFR) C677T polymorphism were found to be associated with increased risk of arterial thrombosis in neonates. [1, 16].

Our patient was found to have swollen, blue left upper limb with skin maceration in the delivery room, physical examination revealed cold cyanotic below elbow left upper limb with skin macerations, absent peripheral pulses, prolonged capillary refill time, and absent Moro and grasp reflexes in the left upper limb. Diagnosis of left subclavian artery thrombosis was established by Doppler ultrasound and left upper limb Computed tomography angiography (CTA) at 6 hours of age.

Investigations for the etiology revealed a normal complete blood count and coagulation profile. Coombs test was negative, CRP was 17.7, and blood cultures showed no growth. Chest and left upper limb x-rays revealed no fractures indicating traumatic delivery. Normal placental examination, normal echocardiography with no evidence of congenital heart disease, normal levels of protein C, protein S, and antithrombin.

Lupus antiphospholipids and anticardiolipin antibodies were negative. Screening for genetic procoagulant mutations was done, including DNA-based assays for ACE I/D, PAI-1 4 G/5G, Factor XIII Val34Leu, HPA1 (1a/1b), Factor V Leiden, and prothrombin G20210A and were all normal.

She was found to be heterozygous for the methylenetetrahydrofolate reductase MTHFR (C667T) polymorphism. Homocysteine level was 7.9 on the 14th day of age. Methylenetetrahydrofolate reductase is an enzyme that catalyzes the reduction of 5, 10-methylenetetrahydrofolate to 5-methyltetrahydrofolate, which is considered to be the primary form of folate in the circulation and an essential co-substrate necessary for methylation of homocysteine into methionine [9]. Decreased enzymatic activity can result in hyper-homocysteinemia, which, in turn, has a deleterious effect on vascular endothelium and promotes thrombotic complications. [9, 12, 13].

Two common polymorphic variants of the MTHFR gene were found to be associated with an increased risk of thrombosis. Both C677T (p.Ala222Val) and c.1286A⟶C (p.Glu429Ala) are polymorphic variants that can produce a thermo-labile enzyme with reduced enzymatic activity. Thus, can result in mild to moderate hyper-homocysteinemia in homozygotes for any of these missense variants [9].

Many studies concluded that 677C > T mutation in the MTHFR gene has been associated with thermo-labile enzyme and elevated homocysteine concentrations in homozygous and heterozygous individuals. [17–19].

Despite the debate regarding the clinical significance of heterozygous 677C > T polymorphism, and its association with intrauterine ischemia, Hakim et al. reported 2 cases of intrauterine upper limb ischemia who were found to be heterozygotes for the 677C>T polymorphism of the MTHFR gene and normal homocysteine level [11].

Alioglu et al. also reported a case of intrauterine right lower limb ischemia, for whom thrombophilia assessment revealed a heterozygous state for the 677C>T variant of MTHFR associated with mild hyper-homocysteinemia [12]. Khriesat et al. also reported a case of intrauterine right upper limb ischemia in a preterm infant who was found to be heterozygous for both MTHFR 677C > T gene mutation and Factor V Leiden gene mutation [13].  Table 1 compares our patient and other patients diagnosed with intrauterine limb thrombosis and were found to be heterozygotes for the MTHFR 677C-T gene mutation.

The management of neonatal arterial thrombosis is debatable and controversial. Monagle et al. study state that the management should be individualized depending on the extent of thrombosis and the urgency of the clinical presentation [20].

Anticoagulation agents are the recommended initial treatment of neonatal atrial thrombosis [21–23]. Heparin can be used in case of clinically significant thrombosis to prevent clot expansion or embolism. However, followed by low-molecular-weight heparin (enoxaparin) is the safest and the most commonly used anticoagulant in neonatal thrombosis and it is indicated for the primary treatment of neonatal thromboembolism [12, 21, 23–26]. Our patient was started on heparin at 4 hours of age and on low-molecular-weight heparin at 46 hours of age.

However, in severe cases and when medical treatment fails, amputation of the affected limb is the last option. Amputation should be delayed as long as possible. A multidisciplinary team of pediatricians, orthopedic surgeons, vascular surgeons, and physiotherapists should be involved in the management of such cases to provide the best outcome for the patient [27, 28].

Our patient developed wet gangrene (Figure 3). Orthopedic and vascular surgeons were consulted. Left upper limb amputation was done after the failure of medical management. Two of the reviewed cases in the table (Table 1.) retained perfusion, either after thrombolytic therapy or after balloon angioplasty [11, 12].

## 4. Conclusion

This is one of the few reported cases of an unusual presentation of thrombophilia presenting at birth with intrauterine limb thrombosis, who unfortunately ended with limb amputation. Intrauterine limb ischemia of the limbs is a rare neonatal condition that presents a significant functional and vital risk. We recommend investigating neonates and their parents for complete thrombophilia mutations when they present with unusual vascular occlusion sites as newborns.

## Figures and Tables

**Table 1:** Summary of the clinical findings, laboratory investigations, treatment provided, and outcome in our patient and other patients diagnosed with intrauterine limb thrombosis and were found to be heterozygotes for the MTHFR 677C-T gene mutation.

Report	Our case	Hakim [11]	Hakim [11]	Alioglu [12]	Khriesat [13]
Gender (M/F)	F	M	M	M	M

Gestational age	Full-term	Premature/34 weeks gestation	37 weeks gestation	38 weeks gestation	33 weeks

Mode of delivery	NSVD	Cesarean section due to previous C/S and fetal distress	Cesarean section	NSVD	Cesarean section due to fetal distress

Birth weight	3665-g	4750-g	3060-g	4500-g	1950-g

Affected limb/artery CTA/doppler US	Left upper limb/left subclavian artery	Right axillary arterial thrombosis extending over 2 cm with no filling of the distal arteries	Right upper limb/Right subclavian artery	Right leg/iliofemoral arterial thrombosis	Right arm/absence of flow in the right brachial and radial arteries

Findings	Swollen, cyanosed left forearm, and hand with skin maceration. Absent spontaneous movements, Moro or grasp reflexes	White then cyanotic right upper limb. With an absent Moro reflex on the right side	The ischemic appearance of the right forearm and right hand was noted with areas of skin necrosis and a delimitation line slightly below the elbow	Pallor and coldness of the right leg and a lack of pulse in the right femoral arterial system were detected. The right leg was 3 cm shorter and 3 cm thinner than the left	Pallor and swelling of the right forearm were noticed with a lack of pulse in the right radial and brachial artery and poor movement of the affected limb

Family history and parental consanguinity	Free family history. None consanguineous marriage	No family history of thromboembolism. Mother diabetic for 7 years	A diabetic mother who had been on insulin for several years. There was no family history of thromboembolism	Free family history. No family history of thromboembolism	Free family history. No family history of thromboembolism

MTHFR 677C-T gene mutation	Heterozygous	Heterozygous	Heterozygous	Heterozygous	Heterozygous

Other gene mutations	Free	Free	Free	Free	Heterozygous for Factor V Leiden gene mutation

Thrombophilia screen	Normal protein C, protein S, antithrombin. Negative antiphospholipid and anticardiolipin antibodies	Normal protein C, protein S, antithrombin. Negative antiphospholipid and anticardiolipin antibodies	Normal protein C, protein S, antithrombin. Negative antiphospholipid and anticardiolipin antibodies	Normal protein C, protein S, antithrombin. Negative antiphospholipid and anticardiolipin antibodies	Normal protein C, protein S, antithrombin. Negative antiphospholipid and anticardiolipin antibodies

Homocysteine level	Normal (7.9 *μ*mol/l on 14th day of life)	Normal	Normal	Mild homocysteinemia (21 *μ*mol/l)	Mild homocysteinemia (13.6 *μ*mol/l on 4th day of life)

Treatment given	Heparin loading (75 unit\kg) at 4 hours old, then on heparin drip (28 units/kg/hour) adjusted according to aPTT. Shifted to LMWH after 3 days	Streptokinase thrombolysis was initiated at 17 hours of life, followed by heparin therapy (28 IU/kg/h). Stopped because of GI bleeding and given platelets	Unfractionated heparin curative dose. Stopped after 48 hours due to secondary thrombocytopenia justifying platelet transfusions. Started on streptokinase after improvement in the platelet count	Balloon angioplasty was successfully performed for iliofemoral arterial occlusion. Then, IV streptokinase (2000 U/kg/h after a 4000 U/kg loading dose) and unfractionated heparin (20 U/kg per h) were administered for 5 days	Unfractionated heparin (20 U/kg/h) for 11 days. After the acute period, the patient was treated with LMWH 1.5 mg/kg; two doses/day

Need for amputation	Yes, developed wet gangrene	No, discharged on enoxaparin for 3 months	Failure of the medical treatment and the extension of the skin necrosis, a trans-humeral amputation was done	The patient's perfusion defect improved on day 16 postpartum when he was discharged on LMWH 1.5 mg/kg. Died on postpartum day 28 with an undetermined cause	Right upper limb amputation below the elbow at the age of 20 days. Because the collateral circulation was not established

## Data Availability

The data used to support the findings of this study are available from the corresponding author upon request.
